# Systematic characterization of the barrier function of diverse *ex vivo* models of damaged human skin

**DOI:** 10.3389/fmed.2024.1481645

**Published:** 2024-12-04

**Authors:** Manon Barthe, Laure-Alix Clerbaux, Jean-Paul Thénot, Véronique M. Braud, Hanan Osman-Ponchet

**Affiliations:** ^1^Laboratoires PKDERM, Grasse, France; ^2^Institut de Pharmacologie Moléculaire et Cellulaire, Université Côte d’Azur, CNRS UMR7275, INSERM U1323, Valbonne, France; ^3^Institut de Recherche Expérimentale et Clinique, UC Louvain, Brussels, Belgium

**Keywords:** skin barrier, damaged skin, TEWL, TEER, dermabrasion, tape stripping, inflammation, filaggrin

## Abstract

**Background:**

The skin barrier plays a crucial role in protecting our body against external agents. Disruption of this barrier’s function leads to increased susceptibility to infections and dermatological diseases. Damaged skin can be due to the use of detergents, sunburn or excessive scratching. In the context of the COVID-19 pandemic the recommended hygiene measures to prevent the spread of SARS-CoV-2, such as wearing masks, frequent handwashing, and the use of sanitizers, can also potentially alter the skin barrier.

**Objectives:**

The purpose of the study was to characterize the barrier function of *ex vivo* models of damaged human skin.

**Methods:**

Skin barrier damage was induced through different chemical and mechanical treatments, representative of the potential factors damaging human skin. The skin barrier function was evaluated in terms of permeability, dermal absorption capacity, *stratum corneum* thickness and gene expression of barrier markers. As inflammation is linked to skin barrier integrity, inflammatory markers were also analyzed.

**Results and discussion:**

The different treatments applied to *ex vivo* skin models allow the simulation of diverse degrees of skin damage, making these models valuable for assessing the efficacy of topical products targeted at skin repair and for studying the effects of compromised skin barrier on viral penetration.

## 1 Introduction

The skin, the largest organ of the human body, serves as a vital and multifaceted interface between our internal environment and the external world. The skin serves as a multifunctional barrier, encompassing physical, biochemical, microbiome, and immunological defenses (1–4).

In its role as a physical barrier, the skin’s outermost layer, the *stratum corneum*, is composed of densely arranged dead skin cells called the corneocytes, which are surrounded by lipids and bound together by corneodesmosomes. This well-structured arrangement creates a robust defense against external pressures, including abrasion, cuts, and shocks. The appropriate thickness of the *stratum corneum* is therefore crucial in the barrier function. Moreover, keratinocytes forming tight junctions add an additional layer of defense to the skin’s physical barrier, further enhancing its protective capabilities. The skin’s structural design also plays a crucial role in preventing excessive water loss from the body, thereby averting dehydration and maintaining optimal hydration levels (1, 5). Indeed, in the stratified epithelium of the skin, filaggrin, loricrin, involucrin, and corneodesmosin, are recognized as essential proteins for a functional epidermal barrier. Filaggrin contributes to the structural integrity of the *stratum corneum*. Deficiencies in filaggrin are associated with increased skin pH, potentially hindering repair mechanisms and promoting dehydration. Loricrin constitutes a vital component of the cornified envelope. Reduced loricrin expression compromises the structural integrity of this crucial barrier. Involucrin plays a critical role in the initial stages of keratinocyte differentiation, influencing the formation of the cornified envelope. Corneodesmosin (CDSN) is an intercellular protein that plays a critical role in maintaining skin barrier function (6, 7). CDSN deficiency resulted in lethal-skin barrier disruption in a mouse model (7) and enhanced viral penetration in an organotypic skin model (8). These essential proteins for epidermal barrier function act in concert to ensure a robust epidermal barrier, safeguarding the underlying tissues.

The skin’s immune barrier is composed of two primary types of immune cells: innate immune cells and adaptive immune cells. Among them, keratinocytes and Langerhans cells play crucial roles as key components of the skin’s innate immune system. They actively participate in protecting the skin from potential infections and contribute to its overall immune surveillance. In response to skin barrier damage resulting from injury or exposure to pathogens, keratinocytes and Langerhans cells trigger an innate immune response. They release various antimicrobial peptides and cytokines that further strengthen the skin’s defense mechanisms against infections, providing an essential line of protection for the body (4, 9, 10).

Recently, the recommended hygiene measures to prevent the spread of SARS-CoV-2, such as wearing masks, frequent handwashing and the use of sanitizers have been blamed for altering the skin barrier integrity (11). Repeated exposure to soaps, surfactants, detergents, and high-concentration ethanol disrupts the skin’s natural barrier, leading to loss of the lipid barrier of the *stratum corneum*, protein denaturation, and changes in keratinocyte cell membranes (12). This weakens the skin barrier, causing increased transepidermal water loss (TEWL) (13), greater irritant and allergen penetration, and an elevated risk of skin lesions and inflammation, potentially increasing the risk of bacterial and viral transmission through the skin (14–16). In the context of the COVID-19 pandemic, understanding the role of the skin barrier in human health has gained paramount importance.

Previous studies already showed the impact of various stressors on skin barrier function, such as the tape stripping technique (17–19), sodium dodecyl sulfate (SDS) treatment (20–23), microneedles (24–27), and abrasion (19, 28, 29). But so far, no studies have investigated the impact of mask wearing and frequent handwashing on human skin barrier. In addition, previous studies have primarily examined isolated aspects of skin health and function.

Here we aim to develop *ex vivo* models of human skin damaged by different treatments. We used both chemical treatments (SDS and ethanol solution) and mechanical treatments (tape stripping, dermabrasion using skin preparation pad, and application of surgical mask). Then we aim to systematically characterize the barrier integrity via an array of relevant parameters, such as the permeability, dermal absorption capacity, *stratum corneum* thickness and gene expression of barrier function markers. As inflammation is linked to skin barrier integrity, inflammatory markers were also analyzed. Having a range of well characterized degrees of skin damage models could be instrumental to assess the efficacy of topical products targeted at skin repair or to evaluate the effects of damaged skin barrier on viral penetration.

## 2 Materials and methods

### 2.1 Human skin samples

This study uses anonymized skin samples obtained as a byproduct from Sterlab (France). French Law No. 2004–801 on bioethics did not require the study to be reviewed or approved by an ethics committee because the anonymized nature and byproduct status of the samples.

Skin samples from abdominal plastic surgery were used through the study. Detailed information about the donors can be found in Supplementary Table S1. Skin samples, collected within 24–48 h after surgery, were used in experiments focused on measuring skin barrier integrity and the mRNA expression of biomarkers. For the evaluation of dermal absorption of Lucifer yellow, *stratum corneum* thickness and TEWL measurements, skin samples stored frozen at −20°C were used. Prior to conducting the experiments, any excess of subcutaneous fat was meticulously removed, and skin disks of approximately 2 cm^2^ were carefully excised, washed in a saline solution, and dried using sterile gauze.

### 2.2 Treatments inducing skin damage

Sodium dodecyl sulfate (SDS) solution was used as a chemical treatment approach to disrupt the integrity of the skin barrier. A 3.5% SDS solution (25 μL) was applied to the surface of skin samples placed in culture inserts for 4 h within a humidified cell incubator at 37°C and 5% CO_2_. Subsequently, the skin surface was thoroughly washed to remove any excess of SDS solution, and the incubation was pursued for an additional 20 h. Further information regarding the optimization of treatment conditions with SDS can be found in Supplementary Data S1.

Tape stripping and dermabrasion techniques were used as mechanical methods to partially disrupt the skin barrier. For tape stripping, a 19-mm-wide adhesive tape (Scotch® transparent tape 550, 3 M, France) was used. A total of 10 tape strips were applied, with each strip assumed to remove one layer of the *stratum corneum*. Dermabrasion of skin samples was performed using 10 applications of the Ambu® Unilect™ 2,121 M skin preparation pad (Ambu A/S, Ballerup, Denmark) with a grain diameter of 58.5 μm. Following the tape stripping and dermabrasion procedures, 14 mm diameter skin biopsies were carefully positioned into culture inserts with a surface area of 1.13 cm^2^, specifically designed for 12-well plates (12-well Cell Culture Insert 0.4 μm PET translucent, CellQART – SABEU, Northeim, Germany). These inserts, containing the skin samples, were then carefully placed within a 12-well plate filled with 500 μL of culture medium (DMEM medium supplemented with 10% fetal bovine serum, 50 IU/mL penicillin, and 50 μg/mL streptomycin). The incubation process was carried out for 24 h within a humidified cell incubator at 37°C and 5% CO_2_. Additional data are provided in the Supplementary Data S2.

To simulate the effects of hygiene measures on viral transmission, we employed two approaches: ethanol and surgical mask treatments. Following World Health Organization (WHO) guidelines for effective viral elimination, we applied 25 μL of 80% ethanol solution on the surface of skin samples in culture inserts for 24 h. For the surgical mask simulation, 12 mm diameter punches were used to create mask disks. The mask disks were applied directly on the skin surface in culture inserts for 24 h. To mimic the occlusive effect of wearing a mask, a 12 mm diameter metal ring was placed on top of the mask disk. Skin samples were incubated at 37°C and 5% CO_2_ under humidified conditions.

### 2.3 Treatment with skin barrier repair product

The marketed skin barrier repair product is formulated to promote epidermal repair following various skin conditions such as post-stitches, peeling, or laser treatments. The composition of this product is as follows: Aqua/water, glycerin, dimethicone, panthenol (5%), pentylene glycol, C30-45 alkyl dimethicone, polybutene, sodium citrate, PEG/PPG-18/18 dimethicone, zinc gluconate, madecassoside, dimethiconol, manganese gluconate, sodium hyaluronate, copper gluconate, caprylyl glycol, citric acid, polysorbate 20, tocopherol. Panthenol, also known as vitamin B5, is recognized for its soothing and reparative effects on the skin. Madecassoside serves as a soothing agent that aids in skin renewal. Skin samples in culture inserts were first treated with either SDS solution for 4 h or skin preparation pad then treated with the barrier repair product (referred to as P2) at 10 mg/cm^2^. Skin samples were incubated at 37°C and 5% CO_2_ under humidified conditions for 24 h.

### 2.4 Measurement of skin barrier integrity

The barrier integrity of the *stratum corneum* was evaluated for each skin sample by measuring the Transepidermal water loss (TEWL) and/or transepithelial electrical resistance (TEER). TEWL measurements were performed on skin samples mounted on Franz-type diffusion cells after a minimum of a one-hour stabilization period using a Tewameter® MDD4 and a TM 300 probe (Courage+Khazaka Electronic, Köln, Germany) measuring device. The TEWL results obtained are expressed in g/m^2^/h. TEER measurements were performed on skin samples placed in culture inserts using a Millicell® ERS-2 Volt-Ohm Meter (Merck KGaA, Darmstadt, Germany) and Endohm-24SNAP chamber (World Precision Instruments, Sarasota, United States). The TEER results obtained are expressed in ohm.cm^2^.

### 2.5 *In vitro* dermal absorption of Lucifer yellow

Skin samples were mounted on Franz-type diffusion cells and allowed to equilibrate for 1 h. Glass diffusion cells with an application surface area of 1 cm^2^ and a receptor compartment volume of 3 mL were used throughout the study. The receptor compartment of each diffusion cell was filled with 3 mL of phosphate-buffered saline (PBS). The diffusion cells were placed on a magnetic stirrer. The receptor fluid was continuously stirred at 300 rpm with a small magnetic stir bar. The temperature of the test system was maintained with a water circulating bath set at 37°C to achieve a skin surface temperature of approximately 32°C. Ten microliters of Lucifer yellow solution (10 mg/mL, in PBS) were applied on skin surface for 24 h. At the end of treatment period, the receptor liquid was collected and stored at −20°C pending analysis. On the other hand, the remaining Lucifer yellow on skin surface was removed using five cotton swabs and two strips and skin samples were stored at −20°C. The Lucifer yellow assay was not conducted on the mask and ethanol model due to concerns about skin damage. The prolonged contact time of 24 h with the receptor liquid at 37°C could compromise the integrity of the dermo-epidermal junction, potentially affecting the accuracy of the dermal absorption measurement.

### 2.6 Histological analysis

Skin biopsies were embedded in optimal cutting temperature (OCT) cryo embedding matrix (Thermo Scientific, United States). Cross sections of 5 μm were cut using a cryotome (Cryotome FSE, Shandon Thermo Electron, United States). Skin sections were mounted on glass microscope slides (SuperFrost® Plus, Thermo Scientific, United States) and stained using hematoxylin and eosin. Skin sections were observed using a Nikon Eclipse Ti microscope equipped with a Nikon Digital sight DS-Ri1 high-resolution camera (Nikon Corporation, Tokyo, Japan). The fluorescence related to Lucifer yellow was observed using an FITC filter cube, which displays green fluorescence. Fluorescence quantification was performed using NIS Elements AR software version 5.11.03 (Nikon Corporation, Tokyo, Japan).

To measure the *stratum corneum* thickness, skin sections were treated with a 0.4 N sodium hydroxide solution, which caused the layers of the *stratum corneum* to swell, facilitating measurements. The thickness of the *stratum corneum* was then measured using NIS Elements AR software version 5.11.03 (Nikon Corporation, Tokyo, Japan).

### 2.7 Analysis of Lucifer yellow in receptor liquid samples

The fluorescence emitted by Lucifer yellow was measured at an excitation wavelength of 405 nm and an emission wavelength of 500–550 nm using a GloMax® Explorer plate reader (Promega France, Lyon, France). The measurements were performed in duplicate (*N* = 2) within a black 96-well plate. For determination of Lucifer yellow concentration in the experimental samples, a calibration curve was used with a range of concentrations between 2.5 ng/mL and 2,500 ng/mL.

### 2.8 Measurement of the mRNA expression of barrier function and inflammatory markers

Skin biopsies were cut into small pieces and placed in a tube containing lysis matrix D (MP Biomedicals, California, United States) and 500 μL of RNA lysis buffer (Promega, Lyon, France). Skin biopsies were then crushed using a FastPrep-24TM 5G homogenizer (MP Biomedicals, California, United States). Skin lysates were recovered, and total RNA was isolated using SV Total RNA isolation system (Promega, Lyon, France) according to the manufacturer’s instructions. RNA concentrations were quantified using a BioPhotometer plus (Eppendorf, Montesson, France).

Five hundred ng of total RNA were converted into cDNA using High Capacity cDNA Reverse Transcription kit according to the instructions provided by the manufacturer (ThermoFisher Scientific, Courtaboeuf, France). Real-time PCR was performed on a 7,500 Real-Time PCR System (ThermoFisher Scientific, Courtaboeuf, France). Validated PCR primers and TaqMan MGB-FAM labeled probes (TaqMan Gene Expression Assays, Applied Biosystems) were used in the study and presented in Table 1. The housekeeping gene GAPDH was used as reference gene to normalize the level of mRNA in the different treatment groups.

**Table 1:** Reference of TaqMan gene expression assays.

Gene	Reference
DEFB1	Hs00608345_m1
FLG	Hs00856927_g1
CXCL8/IL-8	Hs00174103_m1
IVL	Hs00846307_s1
LOR	Hs01894962_s1
TSLP	Hs00263639_m1
GAPDH	Hs02758991_g1

PCR amplifications were performed in a total volume of 25 μL using TaqMan® Universal PCR Master Mix No Amperase® UNG according to the manufacturer’s instructions (ThermoFisher Scientific, Courtaboeuf, France). Target and reference gene sequences were amplified independently in separate reactions and each PCR reaction was performed in triplicate. Thermal cycling parameters were as follows: Polymerase activation (10 min, 95°C) followed by 40 cycles of denaturation (15 s, 95°C) and combined annealing/extension (1 min, 60°C). The PCR fluorescence data were analyzed with 7,500 software (version 2.3, ThermoFisher Scientific, Courtaboeuf, France). The increase in the expression of a target gene was expressed as fold change and calculated as 2^−ΔΔCt^.

### 2.9 Statistical analysis

The statistical analysis was carried out using XLSTAT (Lumivero 2023). A non-parametric Wilcoxon paired samples test or a student’s *t*-test following an *F*-test were used. Regarding gene expression, statistical analysis has been done on the ΔCt values. The null hypothesis, which states that the means are equal, was rejected at a significance level of *p*-value <0.05.

## 3 Results

### 3.1 Effect of damaging skin barrier on transepithelial electrical resistance and on transepidermal water loss

**Figure 1:**
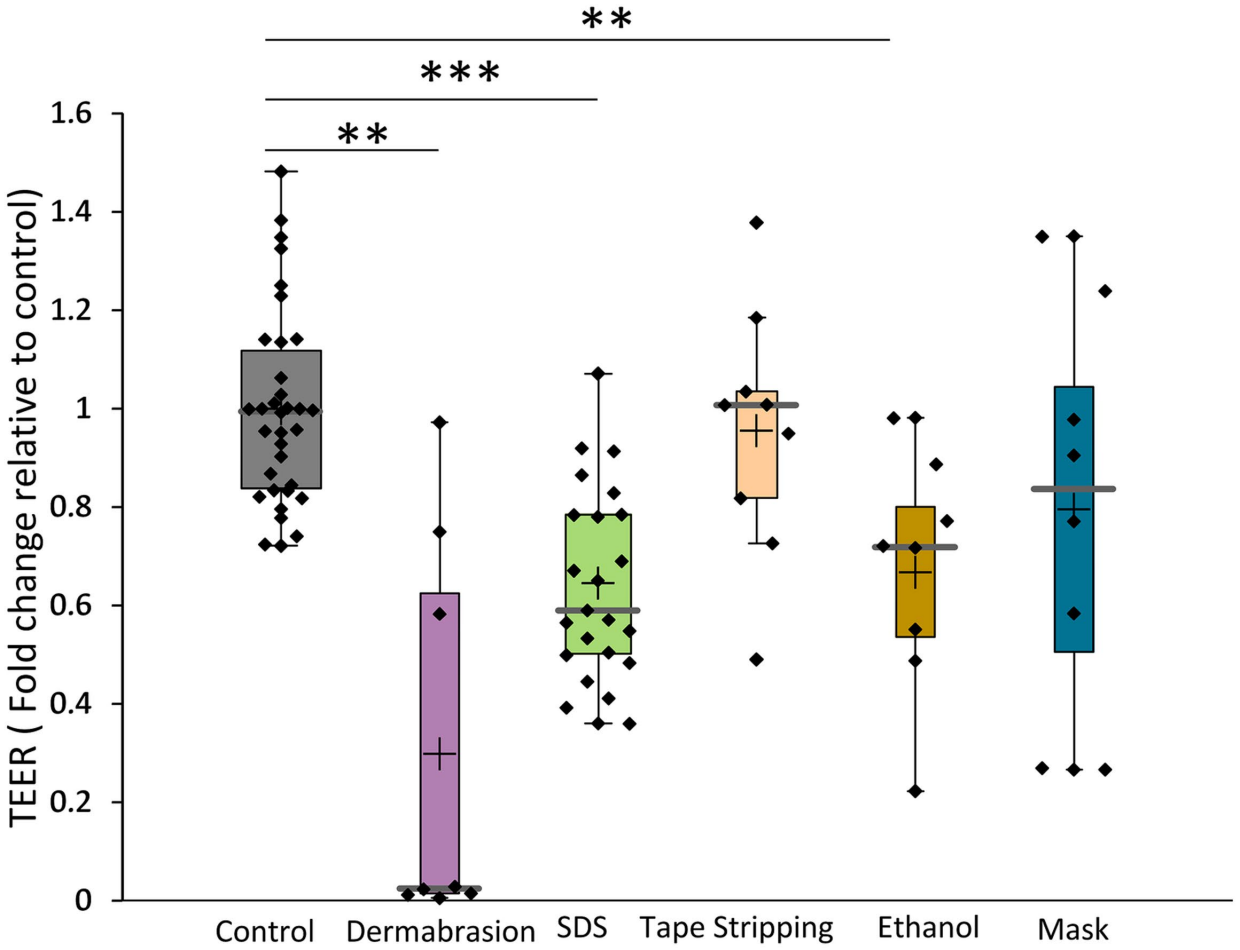
Impact of SDS, dermabrasion, tape stripping, ethanol solution and surgical mask on TEER. The distribution of TEER values for each treatment condition is presented in a boxplot with data point; median (cross), mean (bar) 25th and 75th quartiles (box). *Marks a statistically significant difference compared to control (**: *p* < 0.01; ***: *p* < 0.001). Control (*n* = 34), dermabrasion (*n* = 8), SDS (*n* = 23), tape stripping (*n* = 9), ethanol solution (*n* = 8), and surgical mask (*n* = 8).

The results presented in Figure 1 showed that SDS treatment significantly decreased TEER by 43% (*p*-value = 1.49E^−08^), indicating barrier disruption. Similarly, abrasion with the skin preparation pad resulted in a substantial 98% decrease in TEER (*p*-value =0.004). In contrast, tape stripping did not show a significant effect on TEER, potentially due to its more targeted impact on the *stratum corneum*. TEER measurements further corroborated the impact of ethanol and mask exposure. Twenty-four-hour exposure to 80% ethanol or a surgical mask decreased TEER by 30% (*p*-value = 0.008) and 20%, respectively. These findings suggest a potential for temporary barrier disruption with both ethanol and mask use and highlight the importance of proper mask hygiene and skincare practices during prolonged use.

**Figure 2:**
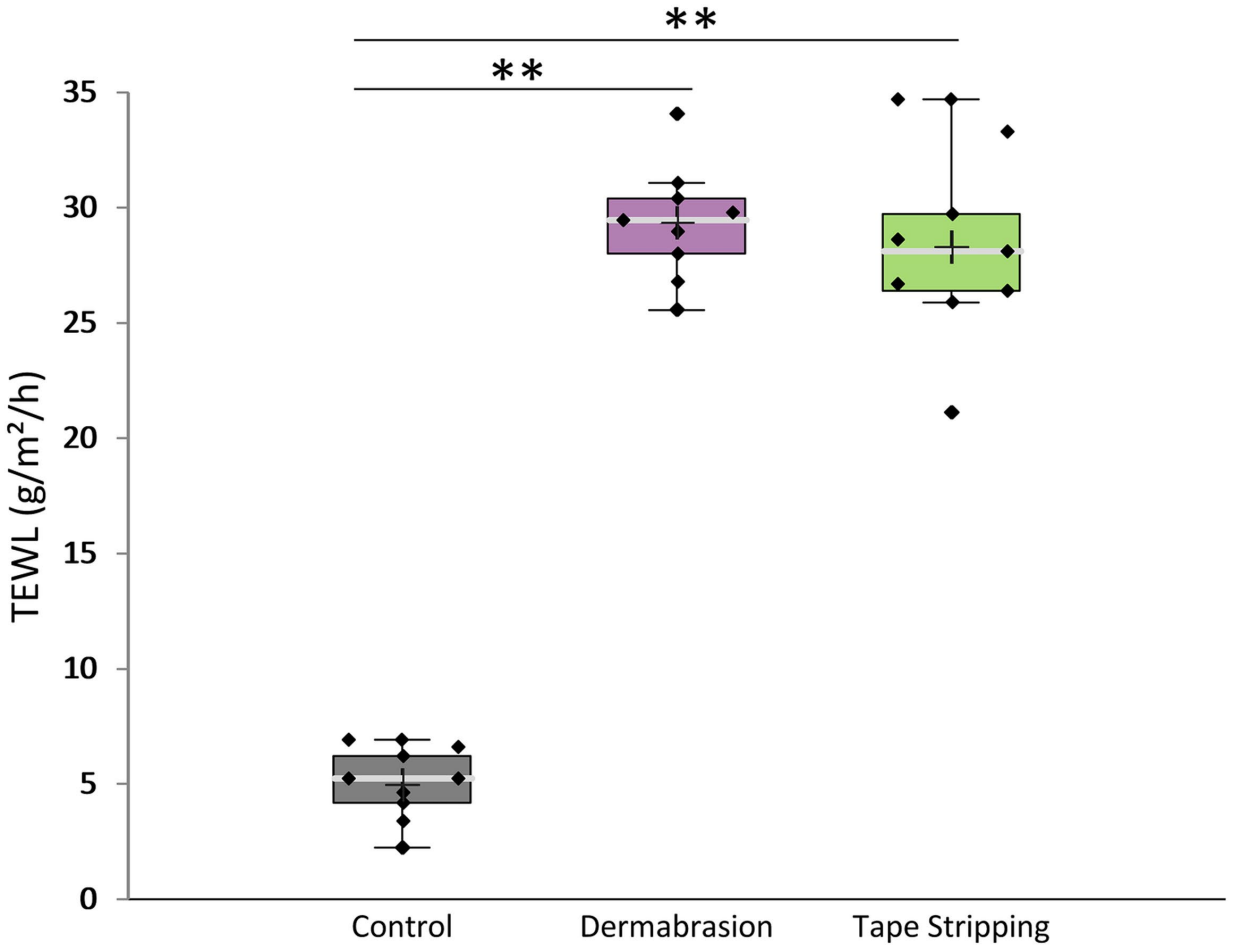
Impact of tape stripping and dermabrasion on TEWL. The distribution of TEWL values for each treatment condition is presented in a boxplot with data point; median (cross), mean (bar) 25th and 75th quartiles (box). **Marks a statistically significant difference compared to control (*p* < 0.01), *n* = 9.

TEWL measurements were conducted only on skin samples that had undergone tape stripping or dermabrasion. Due to the potential for interference from high skin humidity, TEWL measurements cannot be reliably performed on skin samples exposed to SDS, ethanol, or prolonged mask application. The results presented in Figure 2 demonstrated that tape stripping significantly increased TEWL by 5.7-fold (*p*-value = 0.004). Dermabrasion using a skin preparation pad induced a significant 5.9-fold increase in TEWL (*p*-value =0.004). These findings further support the evidence of impaired skin barrier integrity after the use of tape stripping and skin preparation pad.

### 3.2 Effect of damaging skin barrier on *stratum corneum* thickness

**Figure 3:**
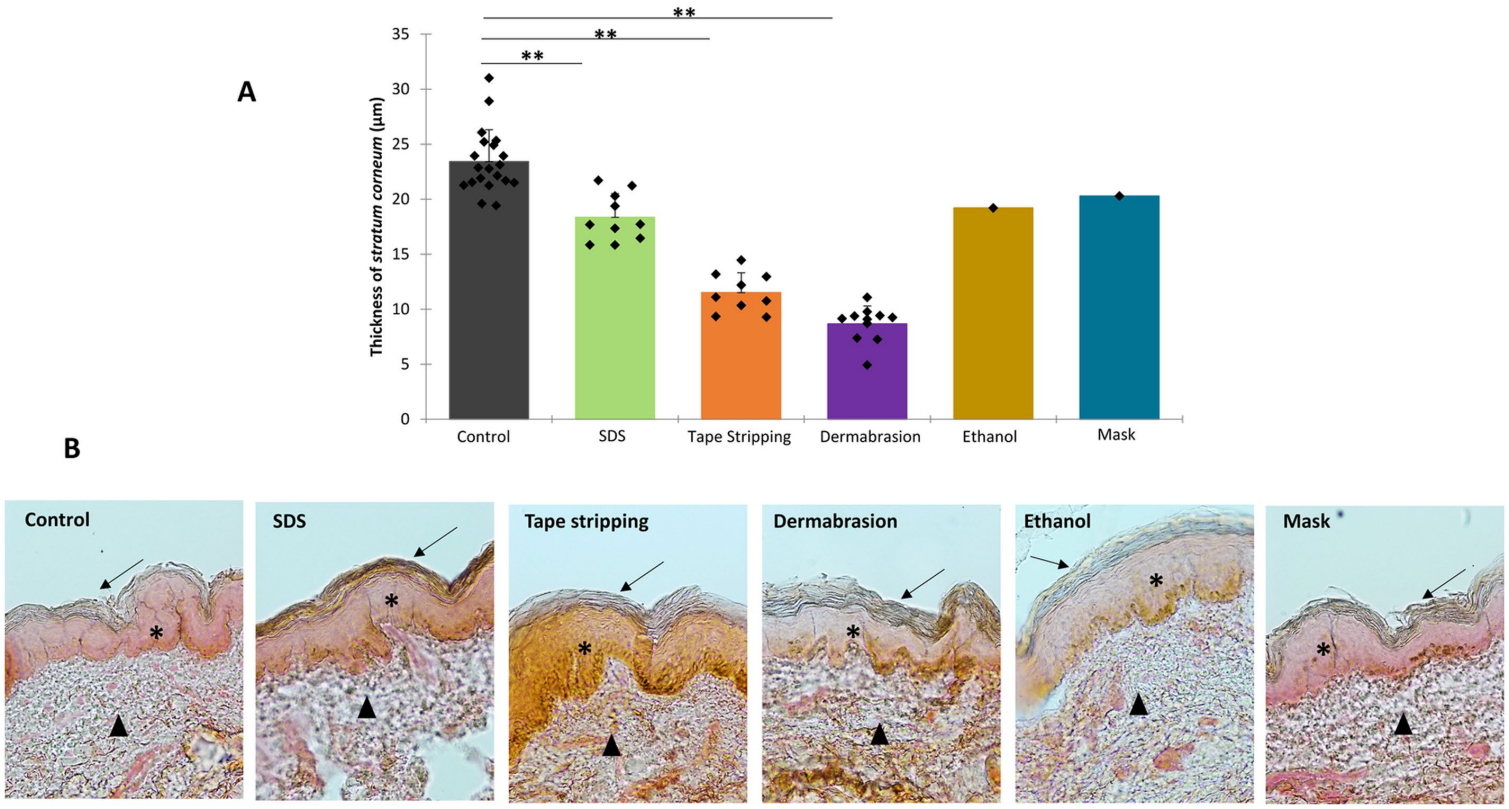
Impact of SDS, tape stripping, dermabrasion, ethanol solution, and surgical mask treatment on *stratum corneum* thickness. **(A)** Distribution of thickness values for each treatment condition presented in a scattergram with datapoint, median (cross), and mean (bar). *Marks a statistically significant difference compared to control, (**: *p* < 0.01). Control (*n* = 20), dermabrasion (*n* = 11), SDS (*n* = 11), tape stripping (*n* = 9), ethanol solution (EtOH, *n* = 1), and surgical mask (*n* = 1). **(B)** Representative images of hematoxylin and eosin-stained skin sections utilized to measure thickness of *stratum corneum* exhibited at X10 magnification. Solid arrow points to *stratum corneum*, star points to epidermis, and solid triangle points to dermis.

The results presented in Figure 3 reveal significant reduction in *stratum corneum* thickness following treatments with tape stripping (−51%, *p*-value = 0.004), dermabrasion (−63%, *p*-value = 0.001), and SDS (−22%, *p*-value = 0.003). Treatments with 80% ethanol solution or surgical mask decreased *stratum corneum* thickness by 18 and 13%, respectively. These findings align consistently with the TEWL and TEER data, collectively demonstrating the impact of all tested methods in compromising skin barrier integrity.

### 3.3 Effect of damaging skin barrier on dermal absorption

**Figure 4:**
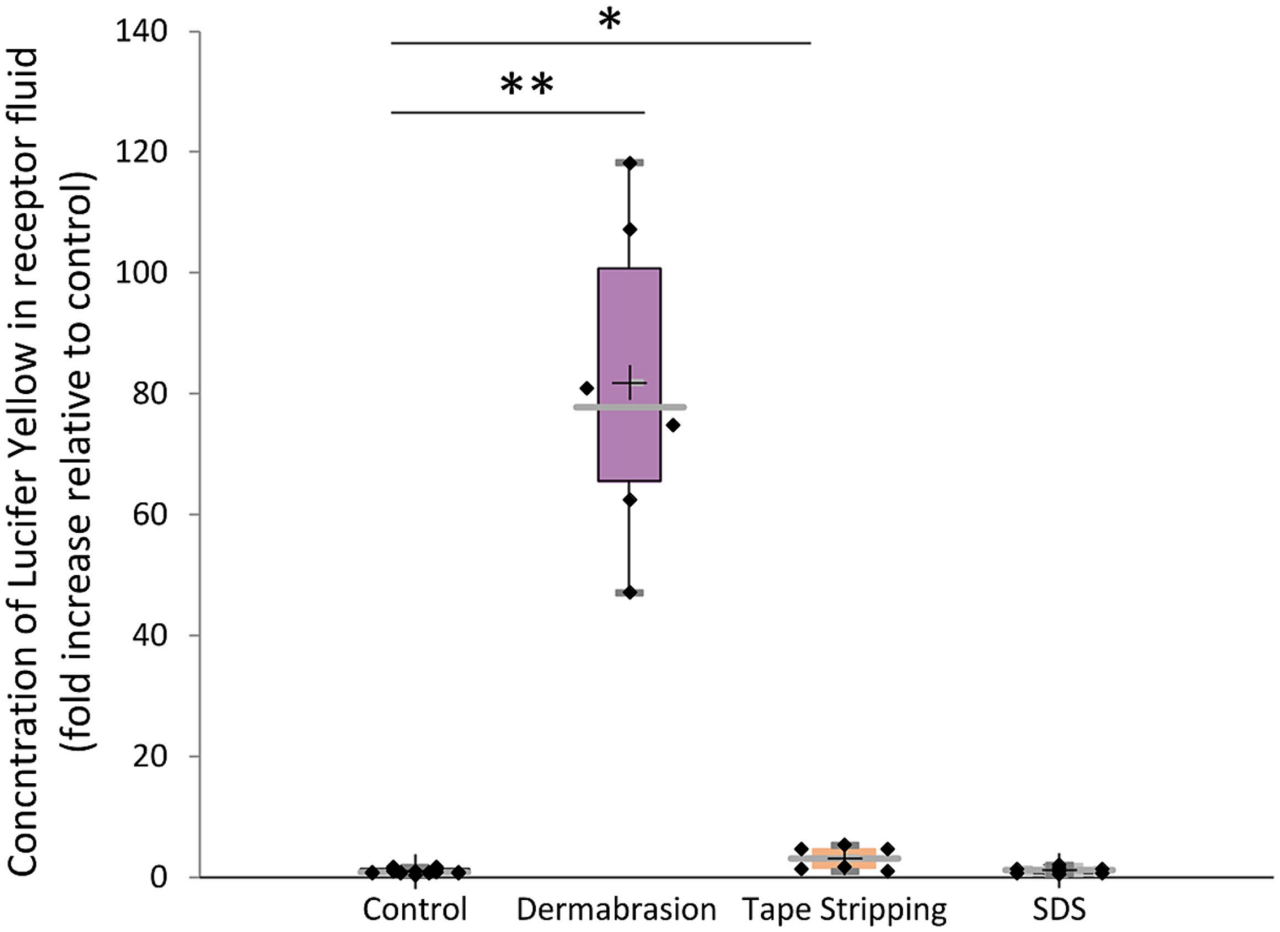
Impact of SDS, tape stripping, and dermabrasion on dermal absorption of lucifer yellow. Concentration of lucifer yellow recovered in receptor fluid after a 24-h treatment period, expressed as fold increase relative to control. The distribution of absorption values for each treatment condition is presented in a boxplot with data point; median (cross), mean (bar) 25th and 75th quartiles (box). *Marks a statistically significant difference compared to control (*: *p* < 0.05; **: *p* < 0.01), *n* = 9.

The analysis of Lucifer yellow in the receptor fluid samples (Figure 4) indicated a slight, non-significant increase of 1.3 times in the concentration of Lucifer yellow after treating the skin with SDS. However, treatment with tape stripping or skin preparation pad led to a significant 3-fold (*p*-value = 0.04) and 133-fold (*p*-value = 0.0007) increase in the concentration of lucifer yellow, respectively.

**Figure 5:**
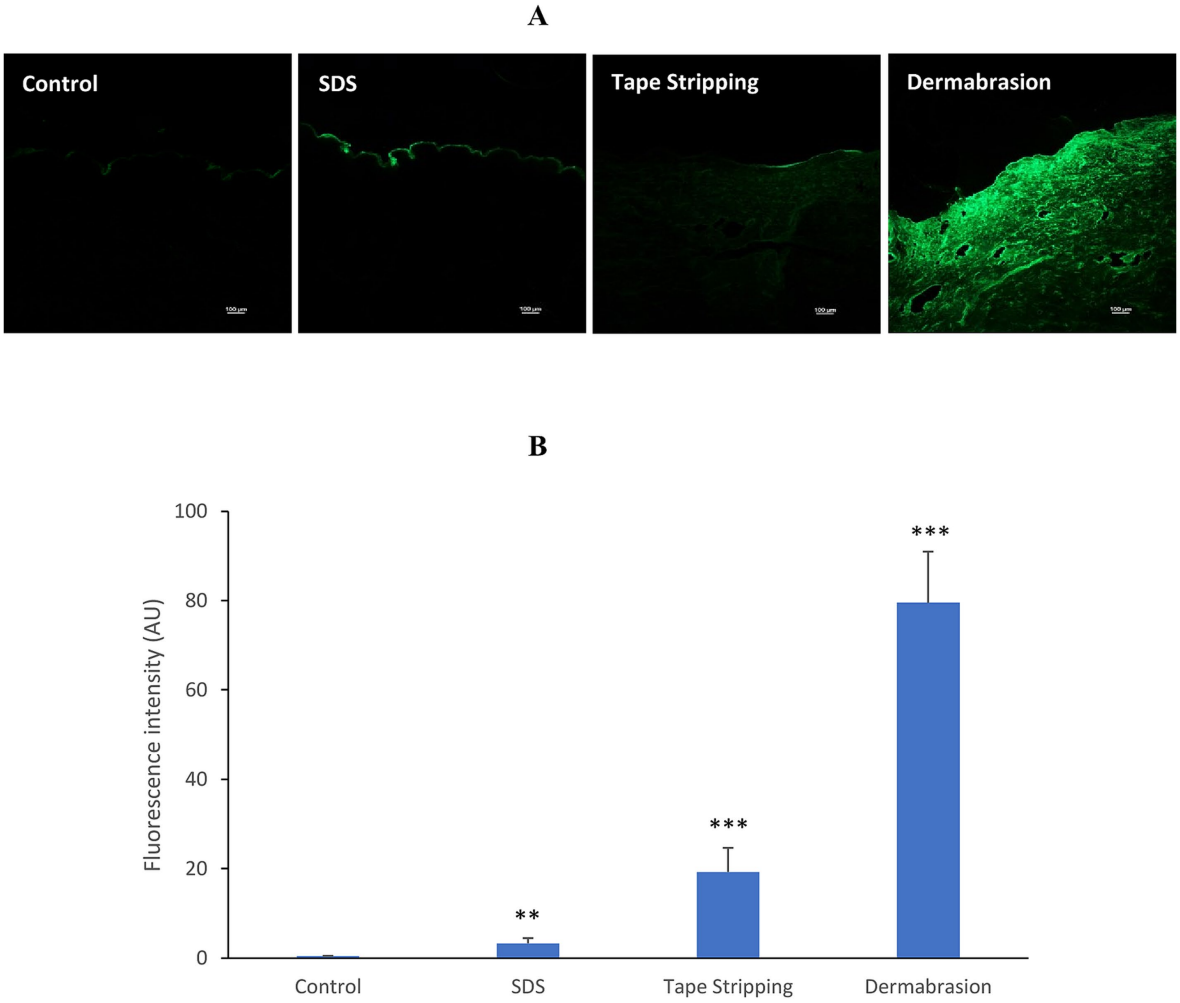
Impact of SDS, tape stripping, and dermabrasion on skin penetration of lucifer yellow. **(A)** Representative images of distribution of lucifer yellow (green fluorescence) in skin sections after a 24-h treatment period. Microscopic images exhibited at X10 magnification. *Ex vivo* human skin samples were pretreated with SDS, tape stripping, dermabrasion, or untreated (control). **(B)** Image analysis of skin sections in control, SDS, tape stripping, and dermabrasion treatment group. *Marks a statistically significant difference compared to control (*: *p* < 0.05; **: *p* < 0.01), *n* = 6–18.

Figure 5A shows the distinct distribution of Lucifer yellow in skin samples following different treatments. SDS treatment primarily confined the fluorescent compound to the stratum corneum, as evidenced by the intense fluorescence signal observed in this layer. In contrast, tape stripping and dermabrasion allowed for deeper penetration of Lucifer yellow, with fluorescence visible throughout the skin sections. While the fluorescence intensity is lower in the case of tape stripping compared to dermabrasion, both treatments facilitated the diffusion of the fluorescent compound beyond the stratum corneum. Quantitative image analysis (Figure 5B) demonstrated a marked 8-fold increase in fluorescence in SDS-treated skin samples compared to the control group (*p*-value = 0.004). Additionally, tape stripping and dermabrasion led to significant fluorescence increase of 45-fold and 185-fold, respectively (*p*-value = 3.81E^-6^).

### 3.4 Effect of damaging skin barrier on mRNA expression of markers associated with skin barrier integrity

**Figure 6:**
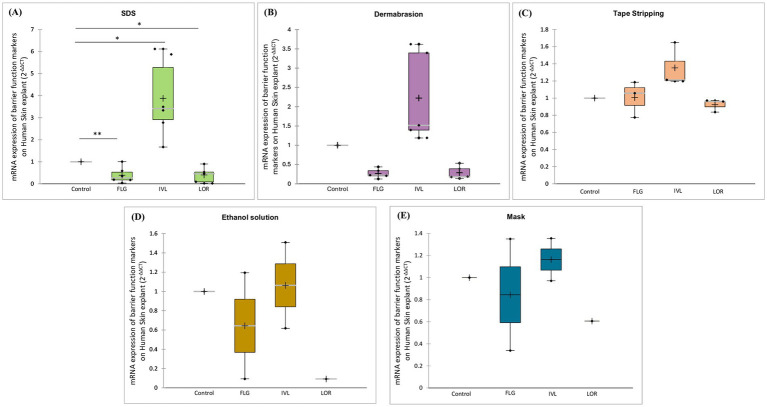
Impact of damaging skin barrier on mRNA expression of barrier function markers. *Ex vivo* human skin samples were treated with **(A)** SDS, **(B)** dermabrasion, **(C)** tape stripping, **(D)** ethanol solution, or **(E)** surgical mask. Untreated skin samples served as control. mRNA expression of filaggrin (FLG), involucrin (IVL), and loricrin (LOR), was measured after a 24-h treatment period using quantitative RT-PCR. *Marks a statistically significant difference compared to control (*: *p* < 0.05; **: *p* < 0.01), *n* = 2–7.

Figure 6 demonstrates the impact of various treatments disrupting the skin barrier on key skin barrier function markers. SDS treatment markedly reduced filaggrin expression by 60% (*p*-value = 0.008) and loricrin by 50% (*p*-value = 0.031), while concurrently inducing involucrin expression fourfold (*p*-value = 0.016), suggesting enhanced keratinocyte differentiation (Figure 6A). Dermabrasion similarly decreased filaggrin and loricrin expression by 79 and 72%, respectively, and increased involucrin expression 2.2-fold, indicating substantial skin barrier disruption (Figure 6B). In contrast, tape stripping had negligible effects on these markers (Figure 6C). Ethanol treatment dramatically decreased filaggrin and loricrin expression by 90% (Figure 6D), while surgical mask usage resulted in a substantial reduction of filaggrin expression by 66% and loricrin by 39% (Figure 6E), highlighting their potential to compromise skin barrier integrity.

### 3.5 Effect of damaging skin barrier on mRNA expression of inflammatory markers

**Figure 7:**
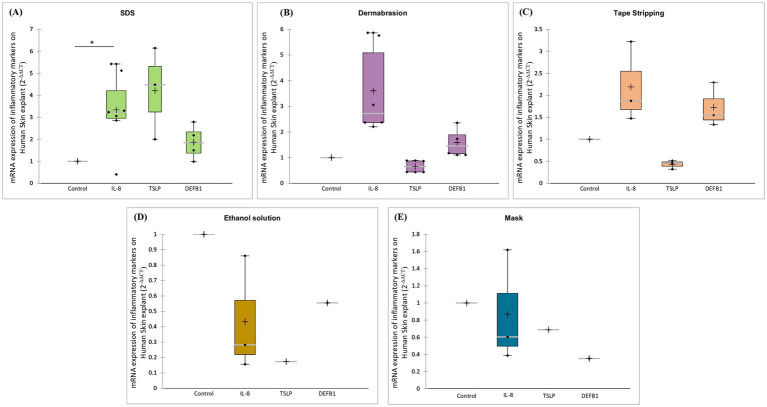
Impact of damaging skin barrier on mRNA expression of inflammatory markers. *Ex vivo* human skin samples were treated with **(A)** SDS, **(B)** dermabrasion, **(C)** tape stripping, **(D)** surgical mask, or **(E)** ethanol solution. Untreated skin samples served as control. mRNA expression of DEFB1, IL-8, and TSLP was measured after a 24-h treatment period using quantitative RT-PCR. *Marks a statistically significant difference compared to control (*: *p* < 0.05), *n* = 2–7.

The results depicted in Figure 7 demonstrate that treatments disrupting the skin barrier trigger an inflammatory response. SDS treatment increased the expression levels of human Beta Defensin 1 (DEFB1) by 1.9-fold, Interleukin-8 (IL-8) by 3-fold (*p*-value = 0.031), and Thymic Stromal Lymphopoietin (TSLP) by 4.2-fold (Figure 7A). Similarly, dermabrasion increased IL-8 expression (3.6-fold) but did not affect TSLP or DEFB1 expression (Figure 7B). Interestingly, tape stripping, while leading to a slight non-significant increase in IL-8, displayed a distinct effect by causing a 57% reduction in TSLP expression compared to control skin (Figure 7C). This suggests a potentially different inflammatory pathway triggered by tape stripping. In contrast, exposure to ethanol solution (Figure 7D) or surgical mask (Figure 7E) did not induce the inflammatory markers IL-8 or TSLP. In fact, there was a trend toward their inhibition, suggesting these treatments may not trigger a significant inflammatory response in the skin.

### 3.6 Effect of barrier repair product on skin barrier parameters

**Figure 8:**
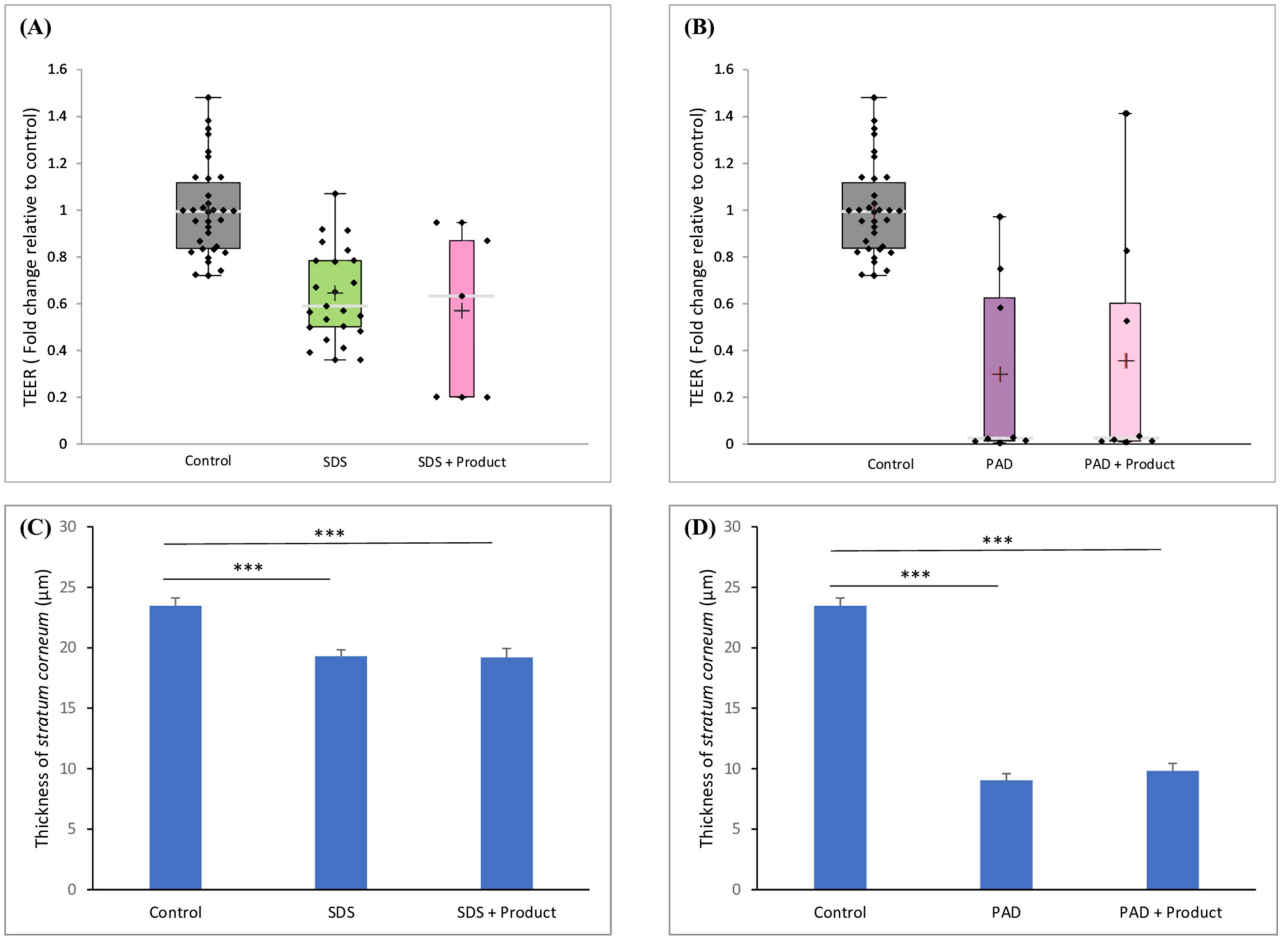
Effect of skin barrier repair product on TEER and on *stratum corneum* thickness. Skin samples were pretreated with **(A,C)** SDS or **(B,D)** dermabrasion using skin preparation pad (PAD) then treated with a skin barrier repair topical product. At the end of a 24-h treatment period, TEER **(A,B)** and *stratum corneum* thickness **(C,D)** were measured. The distribution of TEER values for each treatment condition is presented in a boxplot with data point; median (cross), mean (bar) 25th and 75th quartiles (box). Control (*n* = 34), SDS (*n* = 23), PAD (*n* = 8), SDS plus barrier repair product (*n* = 5), dermabrasion plus barrier repair product (*n* = 8). The data of *stratum corneum* thickness values for each treatment condition is presented mean and SEM. Control (*n* = 21), dermabrasion (*n* = 17), SDS (*n* = 16), SDS plus barrier repair product (*n* = 20), dermabrasion plus barrier repair product (*n* = 20). *Marks a statistically significant difference compared to control (***: *p* < 0.001).

The efficacy of a commercially available skin barrier repair product was assessed by evaluating its ability to restore skin integrity following damage induced by SDS or dermabrasion. No significant improvements in TEER values (Figures 8A,B) or *stratum corneum* thickness (Figures 8C,D) were observed following either treatment regimen, indicating that the product was ineffective in repairing the damaged skin barrier.

**Figure 9:**
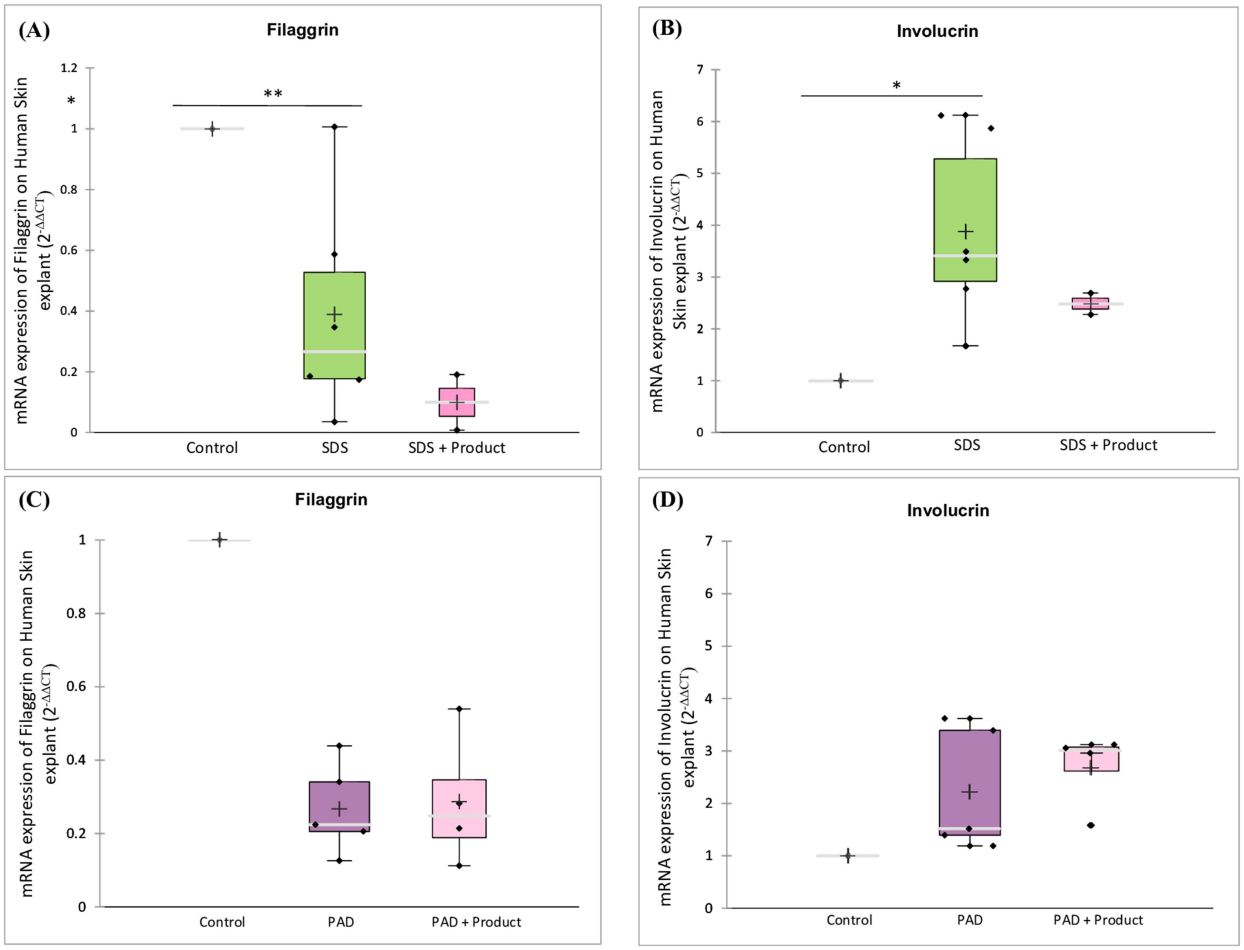
Effect of skin barrier repair product on barrier function markers. Skin samples were pre-treated with **(A,B)** SDS or **(C,D)** dermabrasion using skin preparation pad (PAD) then treated with a skin barrier repair topical product. At the end of a 24-h treatment period, mRNA expression of filaggrin **(A,C)** and involucrin **(B,D)** was measured by quantitative RT-PCR. Control (*n* = 6), dermabrasion (*n* = 5), SDS (*n* = 6), SDS plus barrier repair product (*n* = 2), dermabrasion plus barrier repair product (*n* = 4). *Marks a statistically significant difference compared to control (*: *p* < 0.05; **: *p* < 0.01).

Analysis of barrier function markers (Figures 9A–D) revealed a complex interaction between the skin barrier repair product and different types of skin damage. While SDS treatment alone led to a decrease in filaggrin expression and a compensatory upregulation of involucrin, the combined treatment with the product further impaired the skin’s ability to repair the damaged barrier, resulting in additional decreases in filaggrin and attenuated upregulation of involucrin. In contrast, the product had no significant impact on the expression of these proteins in dermabrasion-damaged skin. These findings suggest that the product’s efficacy may be dependent on the specific mechanisms underlying different types of skin damage.

**Figure 10:**
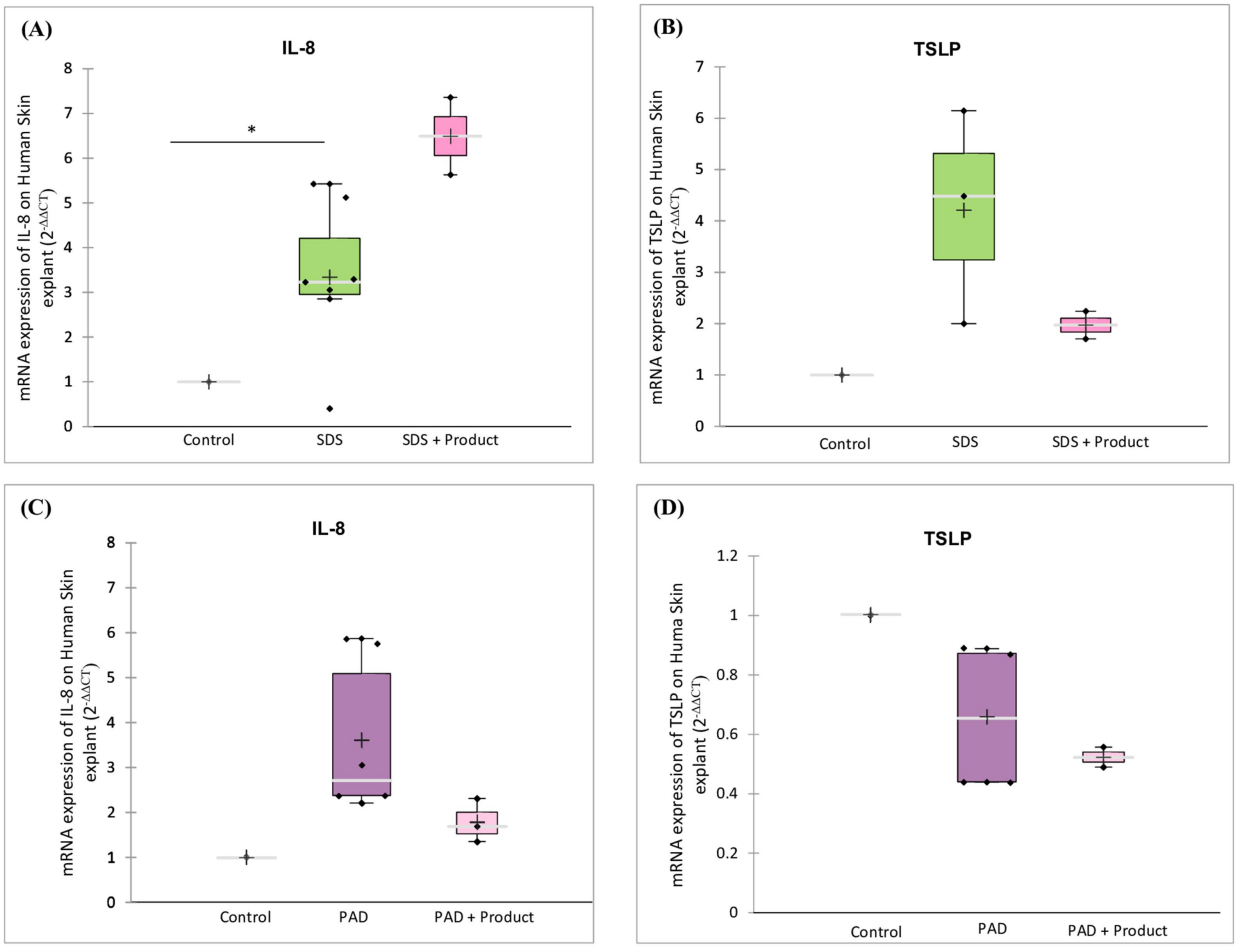
Effect of skin barrier repair product on inflammatory markers. Skin samples were pre-treated with **(A,B)** SDS or **(C,D)** dermabrasion using skin preparation pad (PAD) then treated with a skin barrier repair topical product. At the end of a 24-h treatment period, mRNA expression of IL-8 **(A,C)** and TSLP **(B,D)** was measured by quantitative RT-PCR. IL-8: Control (*n* = 7), dermabrasion (*n* = 6), SDS (*n* = 7), SDS plus barrier repair product (*n* = 2), dermabrasion plus barrier repair product (*n* = 3). TSLP: Control (*n* = 4), dermabrasion (*n* = 4), SDS (*n* = 3), SDS plus barrier repair product (*n* = 2), dermabrasion plus barrier repair product (*n* = 2). *Marks a statistically significant difference compared to control (*: *p* < 0.05).

Analysis of inflammatory markers (Figure 10) revealed that the skin barrier repair product had contrasting effects on SDS- and dermabrasion-induced damage. While there is a trend to increased IL-8 expression (Figure 10A) and decreased TSLP expression (Figure 10B) in SDS-damaged skin, it decreased both IL-8 and TSLP expression (Figures 10C,D) in dermabrasion-damaged skin.

## 4 Discussion

The skin barrier plays a critical role in safeguarding the body from infections and dermatological disorders by acting as a defense against external agents. The emergence of the COVID-19 pandemic has emphasized the importance of maintaining a healthy skin barrier, as a compromised barrier could potentially facilitate viral entry (30–35). With the widespread adoption of hygiene practices during the pandemic to prevent SARS-CoV-2 transmission, concerns regarding potential alterations to the skin barrier have arisen. Therefore, developing models to assess and investigate damaged skin barriers becomes even more crucial.

The primary goal of this research was to establish reliable *ex vivo* models for compromised human skin, offering more ethical alternatives to animal models and aligning with the three Rs principles of replacement, reduction, and refinement in experimental research. Due to substantial physiological differences between animal and human skin, translating animal research findings to humans can be unreliable. Human-based *ex vivo* models developed in this study provide a more accurate representation of human skin, thereby increasing research relevance. These models offer a more accurate representation of human skin function and response to various stimuli compared to animal models.

Transepidermal electrical resistance (TEER) and transepidermal water loss (TEWL) provide complementary insights into skin barrier function. TEER measures the electrical resistance across the skin, reflecting its overall integrity. Conversely, TEWL quantifies water loss through the skin, increasing as the barrier’s integrity weakens. A variety of techniques were used to compromise the skin barrier, including tape stripping for sunburn replication, dermabrasion for procedure-related damage, SDS for induction of itch-like irritation, and simulations of excessive handwashing and mask use.

All treatments significantly compromised skin barrier integrity, as evidenced by changes in TEER and TEWL. SDS, ethanol, and surgical mask reduced TEER, while the skin preparation pad decreased TEER and increased TEWL. Tape stripping primarily increased TEWL without significantly affecting TEER.

The difference between the effects of tape stripping on TEER and TEWL can be attributed to the different properties being measured and the specific mechanisms involved. TEER is a measure of the electrical resistance across a biological barrier, traditionally reflecting the tightness of cell junctions. While tight junctions are not typically found throughout the entire *stratum corneum*, some of their components can be detected in the lower portions of the *stratum corneum*, and occasionally even in the outer *stratum corneum* under certain circumstances. Therefore, when the *stratum corneum* is removed through tape stripping, the electrical resistance may not be significantly affected because TEER primarily depends on the integrity of these tight junction components within the *stratum corneum*. It’s worth noting that tape stripping might not directly disrupt these tight junctions. On the other hand, TEWL measures the rate of water evaporation from the skin surface. When the *stratum corneum* is partially removed, the skin’s barrier function is compromised, allowing more water to evaporate from the skin surface. As a result, TEWL increases after tape stripping, indicating an increase in water loss from the skin.

Our findings are consistent with published data demonstrating the detrimental effects of various treatments on skin barrier integrity. Studies investigating the impact of SDS on the skin have consistently shown its disruptive effects on the *stratum corneum* and barrier function. The significant decrease in TEER observed in our *ex vivo* model following SDS treatment is in line with previous research that highlights the weakening of the skin barrier upon exposure to SDS (36, 37). Likewise, the use of the skin preparation pad in our study resulted in a substantial reduction in TEER and a significant increase in TEWL, confirming its damaging effects on the skin barrier. These results are in agreement with previous investigations that have reported compromised barrier integrity following dermabrasion or similar mechanical interventions (38, 39). In the case of tape stripping treatment, our findings are also supported by previous studies (17, 18, 40).

Our findings suggest a potential for temporary skin barrier disruption with mask wear. Therefore, it is important to consider how mask use compares to clothing in terms of their impact on the skin barrier. Compared to most clothing, masks generally have closer and more continuous contact with the skin. This can lead to increased friction and occlusion, potentially impacting barrier function. This, along with the warm and humid microclimate created by masks around the mouth and nose, could influence the skin microbiome. Clothing generally has a less dramatic effect on microclimate. Moreover, some mask materials may contain chemicals or dyes that could potentially irritate sensitive skin, further impacting the barrier. In contrast, clothing fabrics are typically chosen for comfort and breathability, minimizing these concerns. These factors suggest that mask wear might have a more significant impact on the skin barrier compared to clothing.

Microscopic analysis revealed a significant reduction in *stratum corneum* thickness following SDS, skin preparation pad, tape stripping, ethanol and surgical mask treatments, providing further evidence of compromised skin barrier integrity. Reduction in *stratum corneum* thickness is a well-known feature of barrier impairment, and numerous studies investigating the effects of chemical and mechanical treatments on the skin have extensively documented this phenomenon (19, 20). Moreover, the reduced thickness or the *stratum corneum* also leads to altered tissue integrity. As the outermost layer exposed to the external environment, the *stratum corneum* serves as the body’s primary protective barrier. When this layer is damaged, the skin becomes more permeable (22, 38, 39, 41, 42). Consequently, all methods of damage often lead to increased dermal absorption of molecules, particularly hydrophilic molecules, while lipophilic molecules may show less pronounced increase.

In conclusion, the study highlights that, alongside revealing an increase in dermal absorption when the skin barrier is compromised, the findings emphasize the correlation between the magnitude of this increase and the severity of skin damage. For example, in cases of SDS-induced damage, the level of dermal absorption is comparatively lower than that arising from tape stripping, which in turn is less than the dermal absorption resulting from dermabrasion. Altogether, our study provides robust support for the idea of increased dermal absorption in the presence of a compromised skin barrier.

However, it is important to acknowledge the inherent limitations of our *ex vivo* skin models. One key limitation is the absence of systemic circulation, which distinguishes it from *in vivo* conditions where blood vessels and systemic circulation are present. This absence can influence the distribution of substances and potentially affect skin responses and also cannot recapitulate the recruitment of immune cells from the blood circulation. Furthermore, our models may not fully replicate the complex interactions that occur between skin and the body’s internal systems, which are inherent to *in vivo* conditions.

Importantly, our *ex vivo* skin models have been instrumental in assessing the permeation of substances through the skin, shedding light on dermal absorption mechanisms. This recognition has led regulatory agencies to integrate *ex vivo* skin models into specific regulatory processes, including considerations of bioequivalence waivers. One of the key advantages related to dermal absorption is that our models closely resemble the conditions of diseased skin, where dermal absorption is known to be increased. This aspect holds great relevance in terms of risk assessment when considering systemic exposure to substances applied topically. Understanding how substances permeate damaged skin barriers in these situations can have significant implications for assessing potential systemic exposure and safety profiles.

To further explore the molecular impacts of the damaging treatments, we assessed the expression of markers of barrier function and inflammation. Our results reveal that SDS, dermabrasion, ethanol and surgical mask treatments elicited a significant decrease in filaggrin and loricrin expression. These findings are in line with the observed effects on TEER, TEWL, and *stratum corneum* thickness, further confirming damage to the skin barrier. In contrast, tape stripping had no discernible effect on these markers.

Filaggrin, loricrin, and involucrin represent pivotal proteins with important roles in the formation of the epidermal skin barrier (23, 24). Filaggrin’s critical contribution to *stratum corneum* construction underscores the widespread consequences of its decreased expression, directly impacting the resilience of the skin barrier. Moreover, diminished filaggrin levels could lead to a rise of skin pH, obstructing repair mechanisms and potentially causing dehydration. Loricrin constitutes a crucial element of the cornified envelope. Diminishing the expression of loricrin compromises the structural integrity of this protective barrier. The reduction in the expression of these barrier-related molecules, filaggrin and loricrin, closely mirrors the barrier dysfunction triggered by SDS, dermabrasion, ethanol and surgical mask treatments. Notably, the roles of these molecules extend beyond the results of our study, as they have been implicated in dermatological diseases such as atopic dermatitis and psoriasis, where their dysregulation contributes to compromised skin barriers (23–29). Involucrin acts as a significant biomarker indicating the initial stages of keratinocyte differentiation. Its expression is triggered subsequently to the migration of mature keratinocytes from the basal layer but before the onset of cross-linking in the upper epidermal layer’s envelope. It also plays a critical role in shaping the cornified envelope formation. The marked increase of involucrin expression may provide insight into a compensatory mechanism adopted by keratinocytes in response to the reduced levels of loricrin and filaggrin arising from the different treatments. On a different note, the lack of an apparent impact from tape stripping on these markers could potentially be attributed to the insufficiency of 10 tape strips to induce significant molecular alterations, particularly considering the potentially superficial nature of tape stripping.

Cytokines play a pivotal role in various inflammatory skin conditions. The observed modulation of cytokine expression resulting from the diverse damaging treatments explored in our study underscores the intricate interplay between disruptions in the skin barrier and the innate immune defense mechanisms of the skin.

Of particular significance is the substantial increase in IL-8 expression following the SDS, tape stripping, and dermabrasion treatments. This increase in IL-8 levels signifies that the damaging treatments elicited an inflammatory reaction. IL-8 is a cytokine that plays a central role in skin inflammation. The observed rise in IL-8 levels in our study correlates with prior research that demonstrated elevated IL-8 levels following SDS treatment in human subjects (29).

The effect of the different treatments on Thymic Stromal Lymphopoietin (TSLP) expression presents interesting perspectives. TSLP is a cytokine that plays a pivotal role in the initiation and maintenance of immune responses, particularly within the context of skin inflammation and allergic reactions. The increase in TSLP expression following SDS treatment is of particular interest. SDS, well known for its ability to induce skin irritation as a surfactant, disrupts the skin protective barrier and triggers inflammation. The increased TSLP expression we observed after SDS treatment could potentially serve as an indicator of the immune system’s reaction to the irritation resulting from the compromised integrity of the skin barrier (43). Conversely, the decrease in TSLP expression subsequent to tape stripping and dermabrasion could indicate a potential mechanism through which the skin seeks to regulate excessive immune activation caused by these treatments. This could potentially be a safeguard mechanism to avoid an over-aggressive immune response that might lead to heightened inflammation.

The consistent expression of human beta-defensins (hBD) in suprabasal keratinocytes is also worth mentioning. Beyond their antimicrobial function, these defensins contribute to wound healing, immune cell attraction, and regulation of inflammation (44). The maintenance of adequate regulation of hBD closely correlates with skin barrier integrity. The observed increase in DEFB1 expression in the aftermath of the damaging treatments could indicate the skin’s adaptive responsiveness to the challenges encountered. Collectively, these findings underscore the intricate and multifaceted nature of immune responses triggered by various damaging treatments.

The impact of various treatments on key barrier parameters in *ex vivo* human skin is summarized in Table 2.

**Table 2:** Summary of the specificity of different treatments for damaged skin.

Treatments	Model	TEER	TEWL	*Stratum corneum* thickness	Dermal absorption	Expression of barrier markers	Expression of inflammatory markers
SDS	Detergent use	↓	–	↓	↔	↔	↑
Tape stripping	Sun Burn	↔	↑	↓	↑	↔	↑
Dermabrasion	Excessive scratching	↓↓	↑	↓	↑	↔	↑
Ethanol solution	Hand washing with hydroalcoholic gel	↓	–	↓	–	↓	↔
Mask	Surgical mask	↓	–	↓	–	↓	↔

Finally, we evaluated the efficacy of a commercially available skin barrier repair product in restoring skin integrity following damage induced by SDS or dermabrasion. While the product demonstrated some anti-inflammatory effects, as evidenced by the modulation of the expression of IL-8 and TSLP, it was less effective in repairing the physical barrier. Notably, the product did not significantly improve TEER values or *stratum corneum* thickness, indicating a limited ability to restore the epidermal barrier. Furthermore, the product exacerbated the detrimental effects of SDS-induced damage, as seen by the further reduction in filaggrin and involucrin expression. These findings suggest that some components of the product may interact with SDS and that the product may not be suitable for repairing surfactant-induced damage and could potentially worsen the condition.

While the product exhibited anti-inflammatory properties in both SDS- and dermabrasion-damaged skin, its impact on the physical barrier was less pronounced. This discrepancy highlights the need for further investigation into the product’s mechanism of action and its optimal application for different types of skin damage. To gain a more comprehensive understanding of the product’s efficacy and limitations, future studies should focus on investigating the product’s impact on lipid barrier components, evaluating the product’s long-term effects to determine its sustained efficacy in repairing damaged skin, and exploring its interaction with different types of skin damage.

In conclusion, this study describes the characterization of *ex vivo* models of damaged skin upon different treatment modeling different degrees of skin damage. The *ex vivo* models offer a valuable platform for investigating various aspects of human skin barriers. They are based on well-preserved human skin explants, closely mimicking human skin physiology by preserving the natural structure encompassing both the dermal and epidermal layers, as well as other key aspects of skin structure and function. The approach used in this study highlights the complexity of the skin’s response to damage and the importance of considering both physical and inflammatory aspects when evaluating skin barrier integrity. However, further work is necessary to standardize such *ex vivo* models to ensure they comply with current regulations and facilitate wider adoption. In addition to refining the model, future research should investigate the role of the skin microbiome in maintaining barrier function and its potential influence on susceptibility to microbial infections and other skin diseases.

## Data Availability

The raw data supporting the conclusions of this article will be made available by the authors, without undue reservation.

## Supplementary material

SUPPLEMENTARY TABLE S1 Donor Information for Skin Samples Used in the Study.

SUPPLEMENTARY DATA S2 Development of ex vivo human skin model.

SUPPLEMENTARY DATA S1 Optimization of treatment conditions with SDS solution.
