# Supplementary material for: Systematic characterization of the barrier function of diverse ex vivo models of damaged human skin
Source: Front Med (Lausanne). 2024 Dec 4;11:1481645. doi: 10.3389/fmed.2024.1481645 (PMC11664247; doi:10.3389/fmed.2024.1481645)
Supplement: SUPPLEMENTARY TABLE S1 — Donor Information for Skin Samples Used in the Study. [file Table_1.pdf]

Systematic Characterization of the Barrier Function of Diverse *Ex Vivo* Models of Damaged Human Skin

Manon Barthe[1,2], Laure-Alix Clerbaux[3], Jean-Paul Thénot[1], Véronique M. Braud[2], Hanan Osman-Ponchet[1*]

[1] Laboratoires PKDERM, Grasse, France
[2] Institut de Pharmacologie Moléculaire et Cellulaire, Université Côte d'Azur, CNRS UMR7275, INSERM U1323, Valbonne, France
[3] Institut de Recherche Expérimentale et Clinique, UC Louvain, Brussels, Belgium

Supplementary Material

Supplementary Table S1: Donor Information for Skin Samples Used in the Study.

Skin donor identification	Frozen or Fresh skin	Age	Sex	TEER	TEWL	Biomarker expression	*Stratum corneum* thickness	*In vitro* dermal absorption
**PKD-HS-09**	Frozen	32	F	X				
**PKD-HS-11**	Frozen	45	F		X		X	X
**PKD-HS-17**	Frozen	38	F		X		X	X
**PKD-HS-20**	Frozen	58	F		X		X	X
**PKD-HS-21**	Fresh	58	F	X		X		
**PKD-HS-22**	Fresh	67	F	X		X		
**PKD-HS-23**	Fresh	38	F	X		X		
**PKD-HS-25**	Frozen	40	F		X		X	X
**PKD-HS-29**	Fresh	54	F	X		X		
**PKD-HS-30**	Fresh	34	F	X		X		
**PKD-HS-31**	Frozen	55	F		X		X	X
**PKD-HS-34**	Fresh	67	F	X	X		X	X
**PKD-HS-34**	Frozen	67	F		X		X	X
**PKD-HS-41**	Fresh	55	F	X		X	X	
**PKD-HS-42**	Fresh	37	F	X		X		
