# Supplementary material for: Systematic characterization of the barrier function of diverse ex vivo models of damaged human skin
Source: Front Med (Lausanne). 2024 Dec 4;11:1481645. doi: 10.3389/fmed.2024.1481645 (PMC11664247; doi:10.3389/fmed.2024.1481645)
Supplement: SUPPLEMENTARY DATA S2 — Development of ex vivo human skin model. [file Data_Sheet_1.pdf]

Systematic Characterization of the Barrier Function of Diverse *Ex Vivo* Models of Damaged Human Skin

Manon Barthe[1,2], Laure-Alix Clerbaux[3], Jean-Paul Thénot[1], Véronique M. Braud[2], Hanan Osman-Ponchet[1*]

[1] Laboratoires PKDERM, Grasse, France
[2] Institut de Pharmacologie Moléculaire et Cellulaire, Université Côte d'Azur, CNRS UMR7275, INSERM U1323, Valbonne, France
[3] Institut de Recherche Expérimentale et Clinique, UC Louvain, Brussels, Belgium

Supplementary Material

1 Supplementary Data S1: Optimization of treatment conditions with SDS solution

1.1 Identifying the Optimal Chemical Treatment for Impairing Skin Barrier Integrity

To determine the most effective chemical treatment that impairs skin barrier integrity, a comparison was made between the effects of a 5% SDS solution and a 1% Triton® X-100 solution on transepithelial electrical resistance (TEER) values.

Human skin samples stored frozen at -20°C were thawed and mounted in cell culture inserts within 12-well plates. The samples were treated topically with either SDS solution or Triton® X-100 solution for 24 hours. Untreated skin samples were used as control. The incubations were performed in a humidified cell incubator at 37°C and 5% CO_2. TEER measurements were taken at the end of the treatment period. The experiment was repeated three times on a total of four donors.

The results presented in Figure S1 demonstrate that SDS treatment induced a significant decrease in TEER value (p-value = $1.8E^{-11}$), while Triton® X-100 had no significant effect compared to the control untreated group. As a result, SDS treatment was demonstrated to be more effective in impairing skin barrier integrity and was chosen in the subsequent experiments.

1.2 Determining the Optimal Concentration of SDS and Treatment Duration for Skin Barrier Impairment

The next steps involved determining the concentration of SDS and treatment duration that would induce sufficient damage without being too aggressive. We first focused on establishing the effective concentration of SDS Solution. For this purpose, skin samples were treated with SDS solution at concentrations ranging from 1% to 5% for 24 hours. The experiment was repeated twice, using skin samples from two donors. The results presented in Figure S2 demonstrate a concentration-dependent decrease in TEER values. TEER was significantly reduced starting from 4% SDS solution (p-value < 0.05). For the subsequent experiments, a median concentration of approximately 3.5% was chosen, corresponding to a TEER reduction of about 50%.

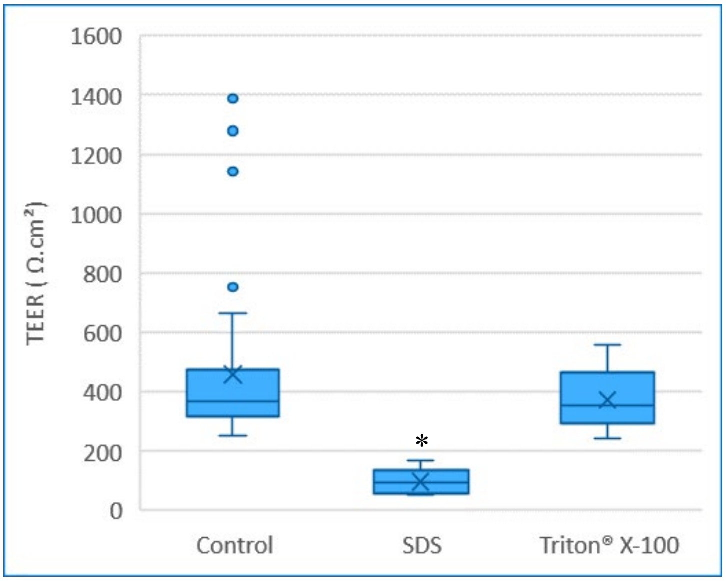

Supplementary Figure S1. Effect of SDS and Triton® X-100 solutions on TEER in *ex vivo* human skin samples. The distribution of TEER values is displayed in a boxplot, featuring the median as a bar, the 25th and 75th quartiles as a box, and the whiskers representing the range between the minimum and maximum values (excluding outlier values). Measurements performed on human skin samples from 4 different donors. Control n=41, SDS n=18, Triton X-100 n=12. * marks a statistically significant difference compared to control (p-value < 0.05), Student's t-test.

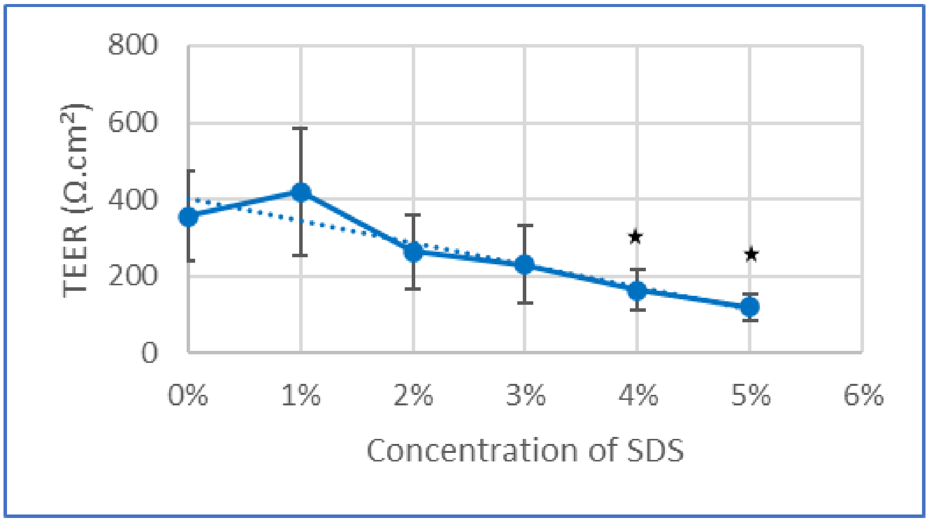

Supplementary Figure S2. Effect of SDS concentration on TEER in *ex vivo* human skin mounted in cell culture inserts. Measurements performed on four skin samples from two donors, n = 4. * marks a statistically significant difference compared to control (p-value < 0.05), Student's t-test.

We then aimed to determine the optimal treatment duration of SDS. Skin samples were treated with a 3.5% SDS solution for durations ranging from 2 hours to 24 hours. The experiment was repeated twice, utilizing skin samples from two donors. The results presented in Figure S3, despite the considerable variability in TEER measurements, reveal that SDS treatment led to a reduction in TEER as early as the first two hours of treatment.

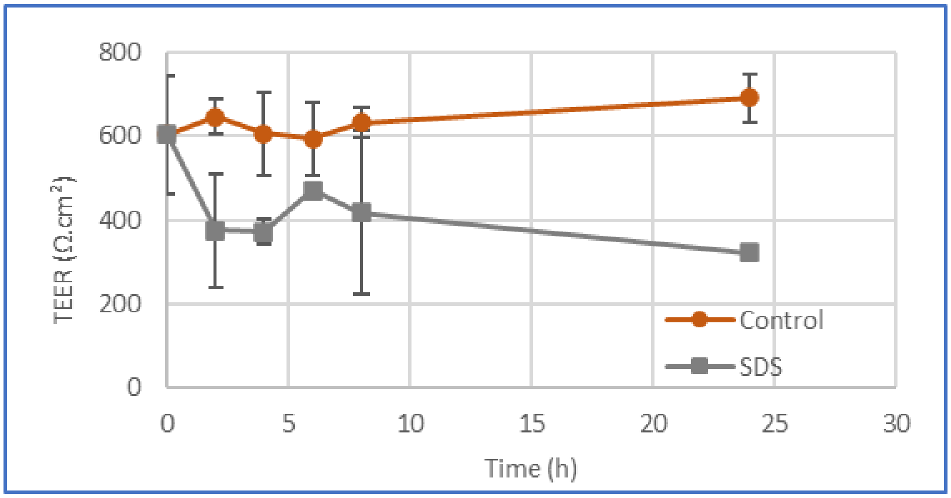

Supplementary Figure S3. Effect of treatment duration with 3.5% SDS solution on TEER in *ex vivo* human skin mounted in cell culture inserts. Measurement performed on four skin samples, n = 4.

Subsequently, to understand the events during the initial two hours of treatment, the experiment was repeated, and TEER measurements were taken at 30 minutes, 60 minutes, 90 minutes, 120 minutes, and 24 hours. The results in Figure S4 clearly show that the maximum effect of SDS on TEER reduction is observed within the first hour of treatment.

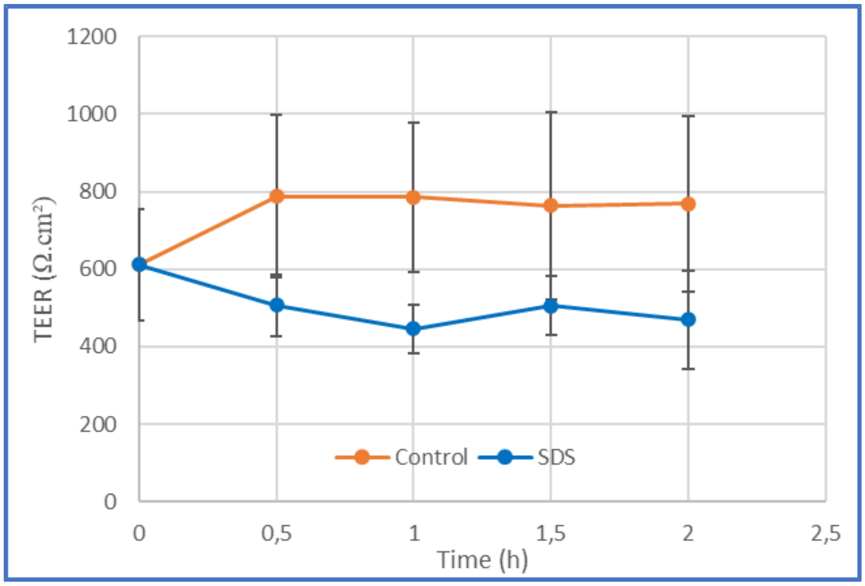

Supplementary Figure S4. Effect of treatment duration with 3.5% SDS solution on TEER in *ex vivo* human skin mounted in cell culture inserts. Measurement performed on four skin samples, n = 4.

1.3 Validation of experimental conditions with SDS on human skin samples Validation of experimental conditions with SDS on human skin samples

This experiment involved the use of fresh skin samples. Skin biopsies, measuring 14 mm in diameter, were placed in cell culture inserts within 12-well plates and treated with a 3.5% SDS solution for 24 hours. Untreated skin samples served as control. The incubations were carried in a humidified cell incubator at 37°C and 5% CO_2. TEER measurements were performed at the end of the treatment period. The experiment was repeated on samples from seven donors, with a minimum of two replicates per donor. The results presented in Figure S5 demonstrated that SDS treatment induced a significant decrease of 43% in TEER (p-value = $1.03E^{-09}$) in fresh human skin samples. A decrease in TEER suggests that the electrical current sent from one electrode to the other is less retained by the tissue. Therefore, this finding provides validation for the treatment conditions with SDS to impairment of skin barrier integrity.

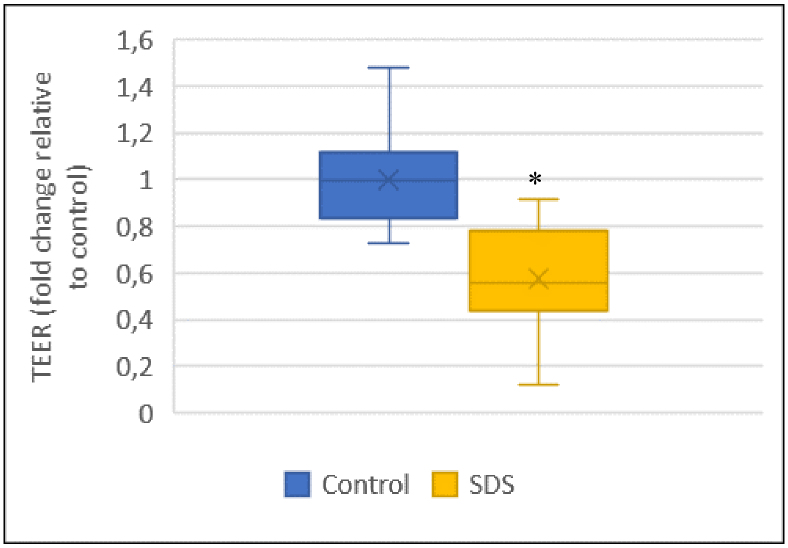

Supplementary Figure S5. Effect of 3.5% SDS solution on TEER in *ex vivo* human skin mounted in cell culture inserts. The distribution of TEER values is displayed in a boxplot, featuring the median as a bar, the 25th and 75th quartiles as a box, and the whiskers representing the range between the minimum and maximum values. Measurements performed on fresh human skin samples from 7 different donors. Control n=14, SDS n=14. * marks a statistically significant difference compared to control (p-value < 0.05), Student's t-test.
