# Supplementary material for: Systematic characterization of the barrier function of diverse ex vivo models of damaged human skin
Source: Front Med (Lausanne). 2024 Dec 4;11:1481645. doi: 10.3389/fmed.2024.1481645 (PMC11664247; doi:10.3389/fmed.2024.1481645)
Supplement: SUPPLEMENTARY DATA S1 — Optimization of treatment conditions with SDS solution. [file Data_Sheet_2.pdf]

Systematic Characterization of the Barrier Function of Diverse *Ex Vivo* Models of Damaged Human Skin

Manon Barthe[1,2], Laure-Alix Clerbaux[3], Jean-Paul Thénot[1], Véronique M. Braud[2], Hanan Osman-Ponchet[1*]

[1] Laboratoires PKDERM, Grasse, France
[2] Institut de Pharmacologie Moléculaire et Cellulaire, Université Côte d'Azur, CNRS UMR7275, INSERM U1323, Valbonne, France
[3] Institut de Recherche Expérimentale et Clinique, UC Louvain, Brussels, Belgium

Supplementary Material

1 Supplementary Data S2: Development of *ex vivo* human skin model

1.1 Set up of the *ex vivo* human skin model

The objective of this work was to develop a reproducible platform that can be adapted for various research purposes. The *ex vivo* model was designed to be cultivated and handled in a sterile manner, allowing for preservation over several days. To establish this model, human skin samples stored at -20°C were used. The approach involved placing a skin biopsy into a cell culture insert, which was then placed in a well of a 12-well plate containing 500 µL of culture medium. Cell culture inserts with a surface area of 1.13 cm² designed for 12-well plates were employed. The process was initiated by obtaining a skin biopsy using a 12 mm diameter punch, matching the size of the insert (Figure S1).

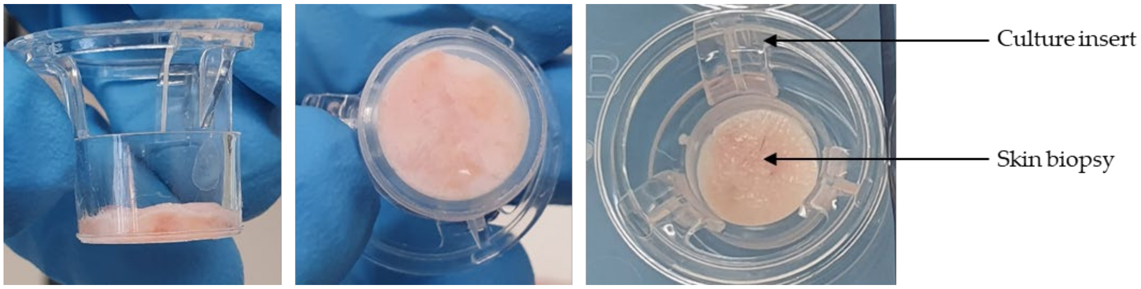

Supplementary Figure S1. Human skin biopsies mounted on cell culture inserts.

Following an equilibration period of at least one hour within a humidified cell incubator set at 37°C and 5% CO_2, the transepithelial electrical resistance (TEER) was assessed at 30-minute intervals over a period of 90 minutes. This analysis was conducted using three skin samples from a single donor. As illustrated in Figure S2, the results underscore that TEER values remained stable across the timeframe spanning from 30 to 90 minutes, approximately at 130 Ω.cm².

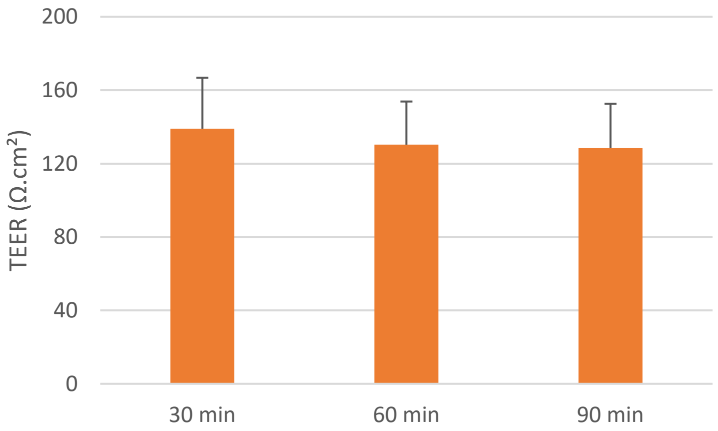

Supplementary Figure S2. TEER values *ex vivo* human skin mounted in cell culture inserts. Measurement performed on three skin samples per condition, n = 3.

To assess the sensitivity of TEER measurements, another experiment was conducted, involving one intact skin sample, one skin sample with the epidermis removed, and one empty insert without any skin sample. The results presented in Figure S3 demonstrate a noticeable variation in TEER values depending on the presence or absence of the epidermal layer and the use of an empty insert. The intact skin exhibited the highest TEER value, indicating a well-maintained skin barrier. In contrast, the TEER value drastically decreased when the epidermis was removed, reflecting a compromised skin barrier. Furthermore, the TEER measurement of the empty insert without skin resulted in the lowest value, suggesting minimal resistance due to the absence of a living tissue barrier. These findings highlight the sensitivity of TEER measurements in assessing the integrity of the skin barrier in different experimental conditions.

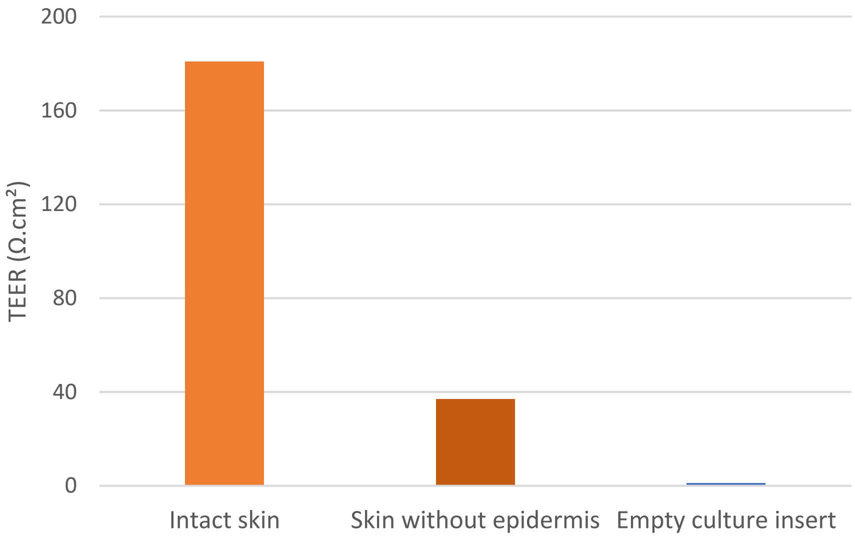

Supplementary Figure 3. Measurement of TEER in intact skin, skin without epidermis and in an empty cell culture insert. Measurements performed on one skin sample per condition, n = 1.

1.2 Control of the tightness of the *ex vivo* skin model

To verify the tightness of the model, a yellow solution of tartrazine was applied to the skin's surface. After 3 hours, the medium beneath the culture insert had turned yellow as can be seen in Figure S4. This indicated that the coloured solution had penetrated the insert, passing between the skin and the culture insert, ultimately reaching the underlying medium. Consequently, it was concluded that the model was not impermeable and, therefore, unsuitable for testing liquid solutions.

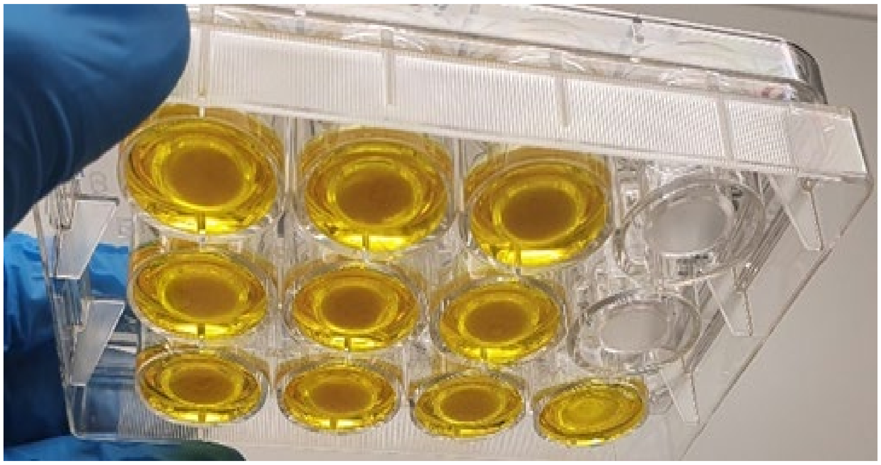

Supplementary Figure S4. Control of the tightness of the *ex vivo* skin model using Tartrazine yellow solution.

Various strategies were employed to ensure the model's impermeability. The initial method encompassed the implementation of seals. One approach explored involved using a syringe piston, incorporating a perforation to facilitate the flow of electric current and the subsequent application of solution (depicted in Figure S5). After applying the yellow dye solution, it became clear that the model was not impermeable, as the medium beneath the culture insert turned yellow (Figure S5).

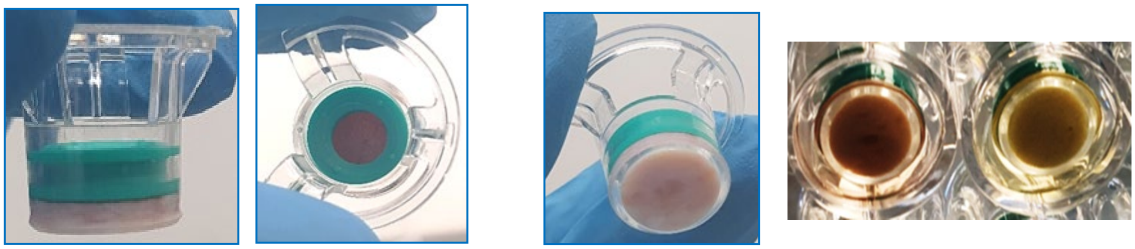

Supplementary Figure 5. Use of syringe piston to ensure the tightness of the *ex vivo* skin model.

Additionally, TEER measurements displayed considerable variability and notably high values (Figure S6). In summary, the utility of the piston syringe approach was found to be ineffective.

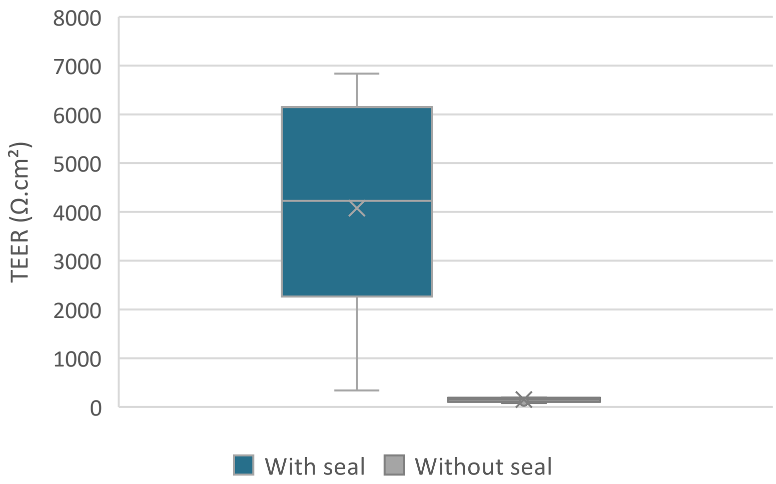

Supplementary Figure S6. Measurement of TEER in *ex vivo* skin samples mounted in cell culture inserts with a syringe piston. Measurements performed on seven samples per condition, n = 7.

TEER measurements were found to be highly sensitive to the pressure exerted by the syringe piston on the skin. As illustrated in Figure S7, negligible TEER values were obtained for empty culture inserts and those with a perforated piston. However, a TEER of approximately 200 Ω.cm² was recorded for skin samples alone. When a perforated piston was placed on the skin, TEER increased significantly to 3,500 Ω.cm². Conversely, non-perforated pistons or excessive skin stretching led to drastically elevated TEER values, reaching around 14,000 Ω.cm².

These results unequivocally demonstrate that the pressure applied to the skin substantially impacts TEER measurements. Consequently, the syringe piston model exhibited poor reproducibility between experiments and operators, rendering it unsuitable for reliable TEER assessment.

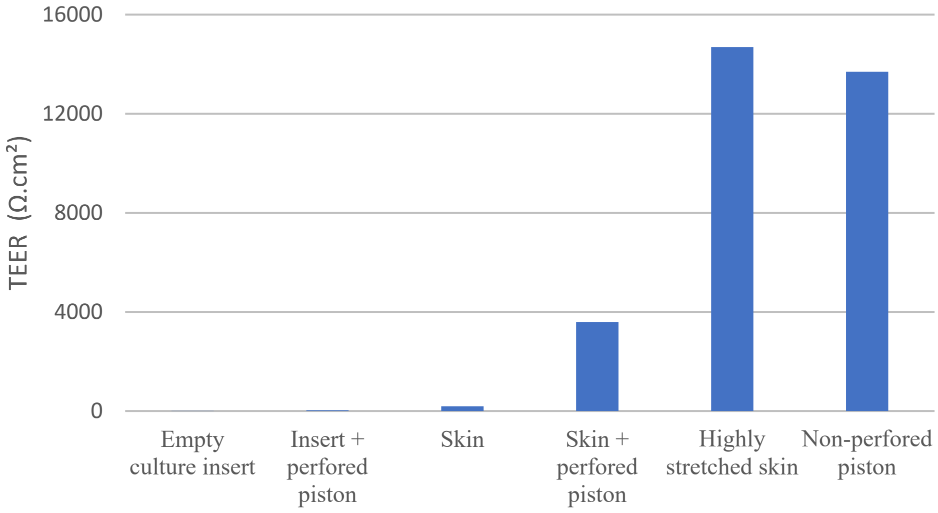

Supplementary Figure S7. Impact of pressure applied to skin samples on TEER measurements. Measurements performed on one sample per condition, n = 1.

Another approach employed to ensure the watertightness of the *ex vivo* skin model involved modifying the seal design, opting for a silicone-based seal that adheres to the skin without applying pressure (Figure S8).

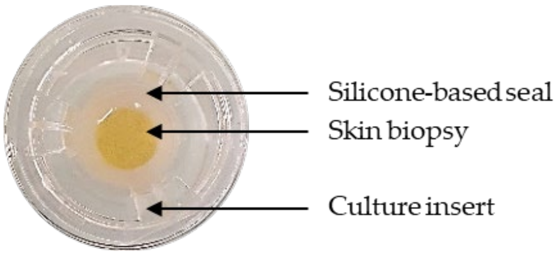

Supplementary Figure S8. Use of silicone-based seal to ensure the tightness of the *ex vivo* skin model.

Skin samples from three different donors were subjected to experiments using cell culture inserts with the silicone-based seal. TEER measurements were conducted at 1, 24, and 48 hours. The TEER values recorded at each time point are presented in Figures S9. The average TEER values were 409, 363, and 333 Ω.cm² at 1, 24, and 48 hours, respectively. The coefficient of variation, which measures variability, remained relatively low at around 25%. Furthermore, upon the application of the yellow dye solution, no leakage of the yellow Tartrazine solution into the medium was observed.

Collectively, the chosen silicone-based seal proved to be advantageous, providing optimal conditions for this reproducible and stable *ex vivo* skin model over time.

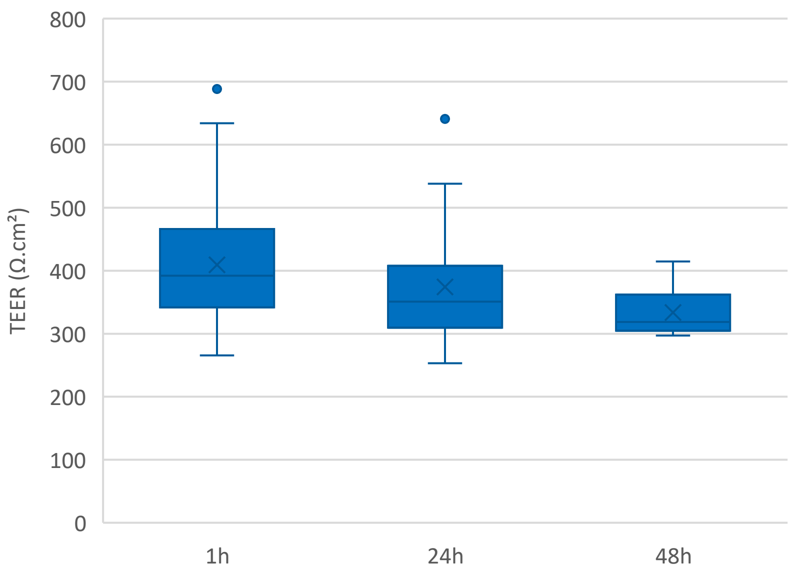

Supplementary Figure S9. Measurement of TEER in skin samples mounted in cell culture inserts with a silicone-based seal. Measurements performed on human skin samples from 3 different donors. 1h: n=25; 24h: n=26; 48h: n=6.
